# Biomimetic Apatite/Natural Polymer Composite Granules as Multifunctional Dental Tissue Regenerative Material

**DOI:** 10.3390/ijms242316751

**Published:** 2023-11-25

**Authors:** Barbara Kołodziejska, Ramona Figat, Joanna Kolmas

**Affiliations:** 1 Department of Pharmaceutical Chemistry and Biomaterials, Faculty of Pharmacy, Medical University of Warsaw, ul. Banacha 1, 02-097 Warsaw, Poland; barbara.kolodziejska@wum.edu.pl; 2 Department of Toxicology and Bromatology, Faculty of Pharmacy, Medical University of Warsaw, ul. Banacha 1, 02-097 Warsaw, Poland; ramona.figat@wum.edu.pl

**Keywords:** dental materials, type I collagen, biomimetic apatite, ibuprofen sodium, drug release, zinc

## Abstract

This study presents a comprehensive evaluation of novel composite biomaterials designed for dental applications, aiming to potentially address the prevalent challenge of dental and periodontal tissue loss. The composites consisted of biomimetic hydroxyapatite (mHA) enriched with Mg^2+^, CO_3_^2−^, and Zn^2+^ ions, type I collagen, alginate, and, additionally, chitosan and sericin. The granules were loaded with ibuprofen sodium salt. The investigation encompassed a morphology characterization, a porosity analysis, a chemical structure assessment, and an examination of the swelling behavior, drug release kinetics (ibuprofen), and release profiles of zinc and magnesium ions. The granules exhibited irregular surfaces with an enhanced homogeneity in the chitosan-coated granules and well-developed mesoporous structures. The FT-IR spectra confirmed the presence of ibuprofen sodium, despite overlapping bands for the polymers. The granules demonstrated a high water-absorption capacity, with delayed swelling observed in the chitosan-coated granules. Ibuprofen displayed burst-release profiles, especially in the G1 and G3 samples. In the case of the chitosan-coated granules (G2 and G4), lower amounts of ibuprofen were released. In turn, there was a significant difference in the released amount of magnesium and zinc ions from the granules, which was most likely caused by their different location in the hydroxyapatite crystals. The cytotoxicity assays confirmed the non-cytotoxic behavior of the biomaterial. These findings suggest the potential applicability of these biomaterials in dental scenarios, emphasizing their multifunctional and biocompatible nature.

## 1. Introduction

The loss of dental or periodontal structures resulting from dental caries, dental anomalies, traumatic injury, periodontal affection, or systematic disease is a very frequent and significant problem in clinical practice. The most common routine challenge in dentistry involves seeking solutions for the regeneration of lost dental or periodontal structures. Therefore, designing new bioactive and biocompatible scaffolds to facilitate the formation of new tissue is one of the current goals of dental tissue engineering. It is also worth adding that the involvement of biomaterials is essential in dentistry, as they provide a porous framework or a carrier for drug delivery to the pivotal target site [1,2,3].

Various natural and synthetic biomaterials, including polymers, ceramics, and their composites, can be used as scaffolds for tooth tissue engineering [4,5,6]. To enhance the biocompatibility of these materials, there is still a demand for scaffolds that mimic the structural, mechanical, and biological properties of natural tissues [7]. It is worth mentioning that bone tissue is a composite material whose main component is type I collagen fibers mineralized with nanocrystalline, multi-substituted, carbonated hydroxyapatite. It also includes other non-collagen proteins and water [8]. Biomaterials imitating this composition could promote interactions and responses between scaffolds and the surrounding environment, thereby enhancing tissue regeneration and repair [9,10].

The most common biomimetic composites consist of type I collagen and hydroxyapatite (Coll/HA). In our previous work, emphasis was placed on obtaining such a composite, with a focus on the ionic modification of apatite to closely resemble biological apatite. In general, biomimetic apatite is calcium–phosphate apatite containing numerous ionic “impurities” of biological apatite, mainly carbonate and magnesium ions. Both of these ions also have numerous biological functions; mainly, they are involved in processes related to strengthening and bone remodeling. Mg^2+^ stimulates the growth of osteoblasts, while CO_3_^2−^ increases the solubility of apatite, which enhances bone reformation or turnover [11].

Numerous studies support the suitability of such biomaterials as dental scaffolds, demonstrating their effectiveness in preserving alveolar bone after a tooth extraction [12,13,14,15]. When designing scaffolds, we focus not only on the function that fills the bone space, but also on creating a bioactive matrix, e.g., with an additional function that delivers a drug substance [16,17]. Combining Coll/HA with other polymers, modifying their porosity, and giving them the right 3D shape may significantly impact the drug and ion release profile as well as other physicochemical properties of the biomaterial [18,19].

The most commonly used natural polymer for preparing biomaterials in the form of granules is based on a biocompatible and non-toxic alginate core [20]. Alginate, a naturally occurring polysaccharide found in algae, becomes polyanionic in an aqueous solution and can be cross-linked by divalent cations such as Ca^2+^. However, the literature suggests that the combination of alginate with collagen may result in mechanical instability and excessive porosity [20]. To address this, a common approach is to cover the granules with a polycation layer, such as chitosan (CH). CH, a deacetylated form of chitin synthesized by different organisms (crustaceans, insects, algae, fungi, and yeasts), is a biocompatible, biodegradable, and non-toxic material [21]. The presence of this complex is expected to decrease the porosity of the membrane and improve its mechanical properties [22].

Sericin is one of the silk proteins extracted from the cocoons of silkworms of different species [23]. For the last few decades, it has been extensively utilized for biomedical applications due to its numerous beneficial biological and physicochemical properties [20,24]. Sericin is rich in hydrophilic amino acids, facilitating easy absorption and the release of moisture [25]. Additionally, the protein possesses antibacterial, anti-inflammatory, antioxidant, and antitumor properties [26,27,28]. When combined with other macromolecular materials, sericin-enriched biomaterials have proven to be biodegradable and non-immunogenic [29].

Therefore, in this study, new composite biomaterials based on biomimetic HA modified with Zn^2+^ ions as anti-inflammatory and osteogenic agents, along with different natural polymers—collagen, alginate, chitosan, and sericin—were prepared in the form of four types of spherical granules. Subsequently, the resulting materials were enriched with ibuprofen (Ibu), a drug substance known for its potent anti-inflammatory properties [30,31]. The aim was to assess the feasibility of utilizing such biomaterials as local bone drug-delivery systems and to evaluate the impact of individual macromolecules on the release profile. The proposed matrices could serve as promising bifunctional materials, releasing both the drug and ions directly in the infected area for the treatment of inflammation following surgical procedures, especially in dentistry, and for the regeneration of bone defects. The obtained materials were subjected to a physicochemical analysis to evaluate their chemical structure and physicochemical properties. In addition, preliminary in vitro studies were conducted.

## 2. Results and Discussion

### 2.1. Physicochemical Parameters of mHA

A detailed physicochemical analysis of the mHA apatite powder is presented in our previous work [11]. According to the TEM, FT-IR, and PXRD results, mHA was identified as a nanocrystalline hydroxyapatite with crystal sizes not exceeding 20 nm and devoid of other crystalline phases. The magnesium and zinc contents, determined using ICP-OES, were 0.33 ± 0.02% and 3.21 ± 0.03%, respectively. Additionally, the carbonate content was determined based on the FT-IR spectra to be 6.4 ± 0.3%.

### 2.2. Morphology and Porosity of the Composite Granules

#### 2.2.1. Scanning Electron Microscopy

**Figure 1:**
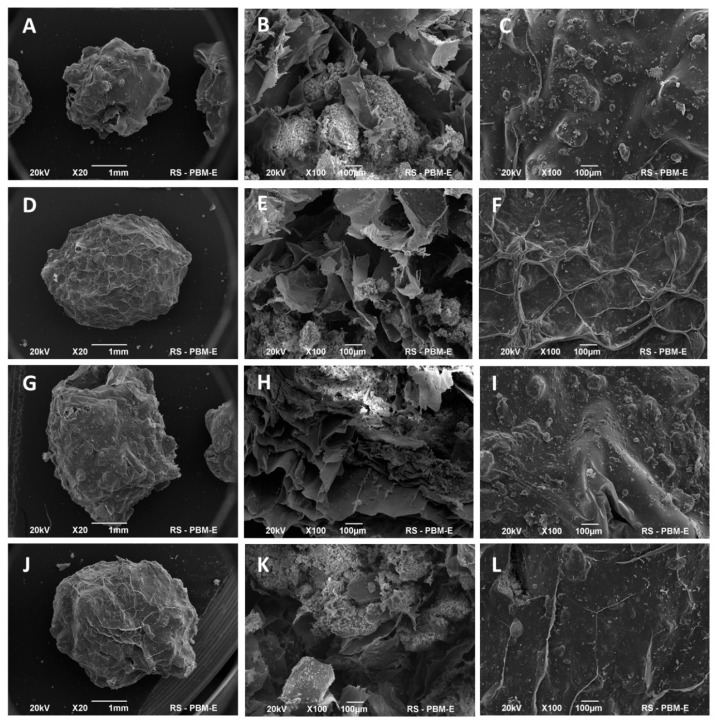
SEM images of G1 (**A**–**C**), G2 (**D**–**F**), G3 (**G**–**I**), and G4 (**J**–**L**) granules. The first column: whole granules; the second column: an internal cross-section; the third column: an outer surface.

The morphological characteristics of the acquired granules were investigated through scanning electron microscopy (SEM), and the findings are presented in Figure 1. The spherical-shaped granules had an approximate diameter of 3.5 ± 0.5 mm. Notably, their surface exhibited conspicuous irregularities, manifesting a pronounced roughness. These irregularities were observed as deep and intricately interconnected cavities, presumably attributable to the alginate through cross-linking processes and the subsequent impact of the freeze-drying procedure. Specifically, the chitosan-coated granules, denoted as G2 and G4, displayed a more uniform and smoother outer surface compared to their uncoated counterparts. The enhanced homogeneity and diminished surface roughness of the chitosan-coated granules were indicative of the efficacy of the coating process. The SEM images obtained from the cross-sections clearly showed the presence of an inorganic fraction (apatite) forming dense agglomerates, as well as a polymer (especially alginate) in the form of a porous structure.

The porosity and irregular structure of the obtained granules are significant in the context of their application as bone substitute material and drug carriers in dentistry. It is well established that the porous nature of biomaterials facilitates cell adhesion, thereby fostering the effective regeneration and reconstruction of mineralized tissue. Moreover, porous structures enable molecular diffusion, which is one of the mechanisms for releasing drug substances from apatite/polymer composites [32,33,34].

#### 2.2.2. Porosity Measurements Using the Brunauer–Emmett–Teller (BET) Method

**Figure 2:**
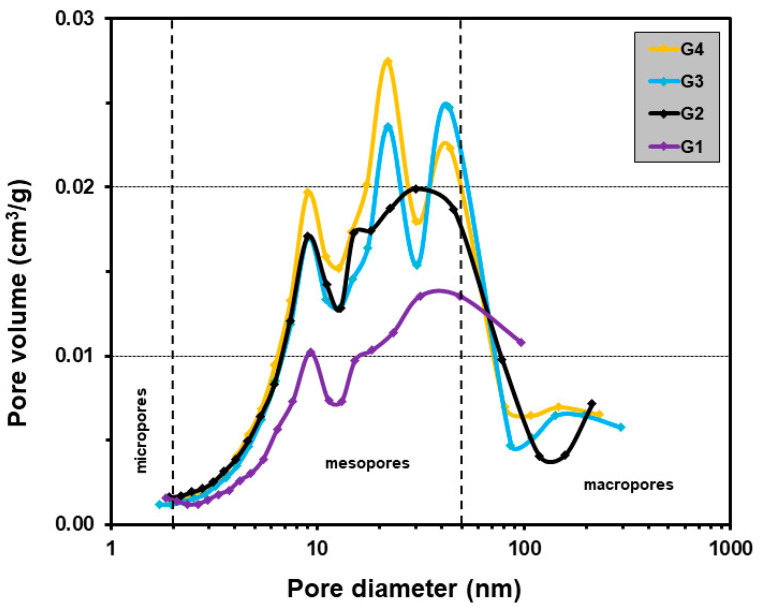
Barrett–Joyner–Halenda (BJH) pore size distribution from 1 to 300 nm based on the desorption isotherms of G1–G4.

The results of the porosity measurements using the Brunauer–Emmett–Teller (BET) method with nitrogen sorption are summarized in Table 1 and Figure 2. All samples exhibited a reasonably well-developed surface, with specific surface area values ranging from 76 to 94 m^2^/g. These findings suggest that the incorporation of sericin and chitosan had only a limited impact on the specific surface area (SSA) value. Notably, sample G1 demonstrated the most advanced surface development, reaching 94 m^2^/g. Furthermore, an examination of the porous structure of the samples revealed a similarity, with mesopores being the predominant feature. This was also confirmed by the shape of the adsorption–desorption isotherms, although specific data are not presented here. The pore volume (V_c_) fell within the range of 0.13–0.23 cm^3^/g. Of particular interest is the observation that sample G1 exhibited the smallest pore volume, indicating that the incorporation of sericin and chitosan contributed to the development of mesoporous structures on the surface. The data in the table show that the addition of sericin or chitosan caused a significant increase in the pore volume, including mesopores.

These findings are particularly relevant in the context of multifunctional biomaterials potentially serving as drug-delivery systems. The degree of porosity and pore size distribution are crucial parameters for drug-delivery systems. The preservation of the original porosity and pore size present in the bone tissue (100–300 μm) leads to the desired material for socket preservation, whereas smaller pores are also required for effective drug loading and release [12,35,36].

### 2.3. Chemical Structure of the Granules

**Figure 3:**
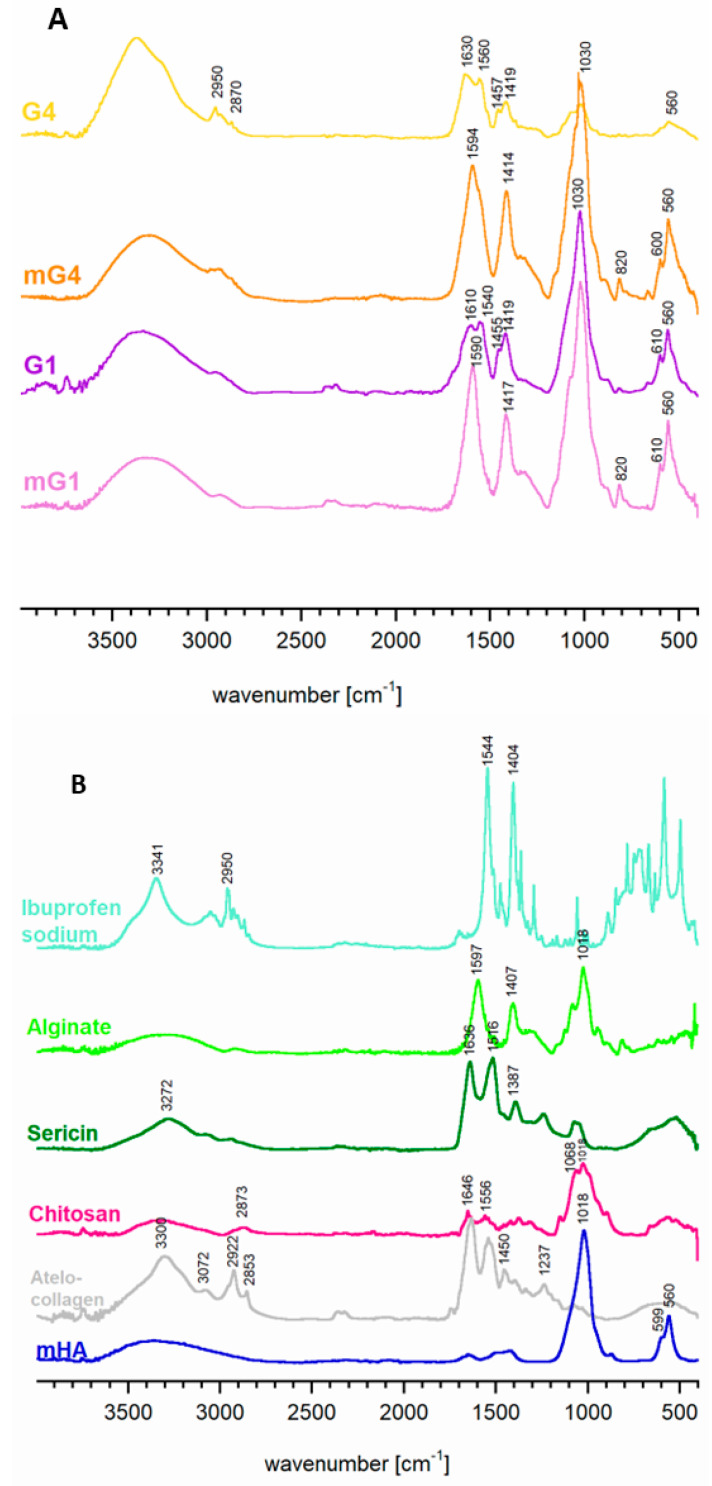
Representative FT-IR spectra of the samples: (**A**) spectra of selected matrix types: mG1 and mG4 and granules with Ibu: G1 and G4; (**B**) spectra of individual granule components.

The FT-IR representative spectra of two distinct matrix types, denoted as mG1 and mG4, along with the corresponding granules (G1 and G4) incorporating the drug substance, are illustrated in Figure 3A. Additionally, Figure 3B displays the spectra of mHA, atelocollagen, alginate, chitosan, sericin, and ibuprofen sodium for a comparative analysis.

The interpretation of the acquired spectra encountered several challenges. Firstly, the polymers within the granules, notably alginate and chitosan as polysaccharides, exhibited spectra that closely resembled each other, as well as the spectra of sericin and type I collagen (proteinaceous materials). Secondly, the bands associated with organic components were broad and substantially overlapped, rendering a comprehensive analysis unattainable. An example of such band overlapping was discernible in the 1540–1630 cm^−1^ range of the spectra for samples G1, G4, G1m, and G4m, representing the bending vibrations of the -NH group. This phenomenon arose from the superimposition of the band at 1516 cm^−1^ and 1636 cm^−1^ originating from proteinaceous sources (sericin and collagen spectra), with bands at 1556 cm^−1^, 1597 cm^−1^, and 1646 cm^−1^, characteristic of alginate and chitosan.

The bands observed in the range of 2870–2950 cm^−1^ originated from the stretching vibrations of -CH groups inherent to both polysaccharides and proteins. Notably, the relative intensity of these bands was more pronounced in the G4 sample, suggesting that their intensity was not solely attributable to the comparatively greater quantity of organic components in the sample, but also to the -CH groups stemming from ibuprofen. In the spectra of the G1 and G4 samples, additional bands at approximately 1540 cm^−1^–1560 cm^−1^ signified the presence of ibuprofen sodium in both materials.

The mHA spectrum was characterized by dominant bands at approximately 1030 cm^−1^ and 600–500 cm^−1^, corresponding to the ν_3_ and ν_4_ vibrations of the orthophosphate groups, respectively. In composite spectra, the ν_3_ band may overlap with the vibrations originating from the C-O groups of the carbohydrate ring (1025 cm^−1^). However, the presence of mHA in the composites was unequivocally confirmed by the 600–500 cm^−1^ bands evident in the G1 and G4 spectra.

### 2.4. Swelling Test

**Figure 4:**
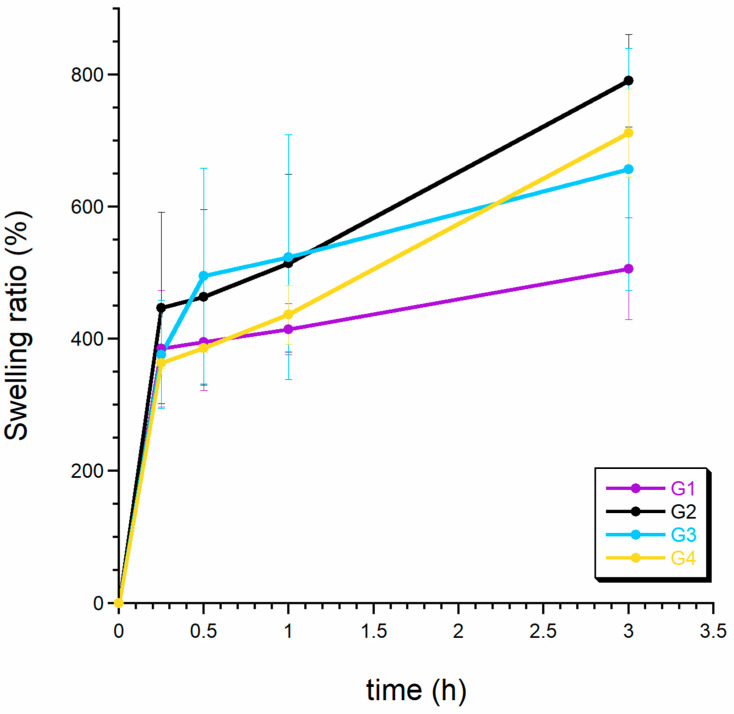
Swelling test. The graph shows the swelling ratio (%) of the granules.

Figure 4 presents the results of the swelling tests conducted on the acquired granules. The outcomes revealed a notably high water-absorption capacity for all the examined samples, which, according to literature references [37,38], can be attributed to the hydration of hydrophilic groups present in chitosan and alginate within the dry granules. It is noteworthy that the water uptake rate exhibited a rapid increase within the initial hour, reaching its maximum in the third hour, which is consistent with the findings reported by other researchers [38].

In general, it was observed that all materials swiftly achieved their maximum swelling ratio (SR). This phenomenon is likely attributable to the foamy and highly porous structure of the matrices, facilitating efficient water penetration [39,40]. Sample G1 displayed the lowest water absorption, potentially linked to its reduced pore volume (refer to the porosity results discussed earlier). This aligns with the established notion that denser materials tend to diminish the water-absorption capacity [35].

An intriguing comparison arose when assessing the swelling capacity of the chitosan-coated granules (G2 and G4). As depicted in Figure 4, both G2 and G4 exhibited relatively modest SR values during the first hour (450% for G4 and 500% for G2). However, by the third hour, they reached the highest SR values of up to 800%.

As previously elucidated, the chitosan membrane may act to mitigate the permeability of the granules. Furthermore, certain hydrophilic groups on the surface of dry calcium alginate granules formed a polyelectrolyte complex with the amino groups of chitosan, resulting in the weakened absorption of water molecules within the polymer network. The subsequent augmentation in granule swelling may be attributed to the degradation of the chitosan coating.

### 2.5. Drug and Ion Release

**Figure 5:**
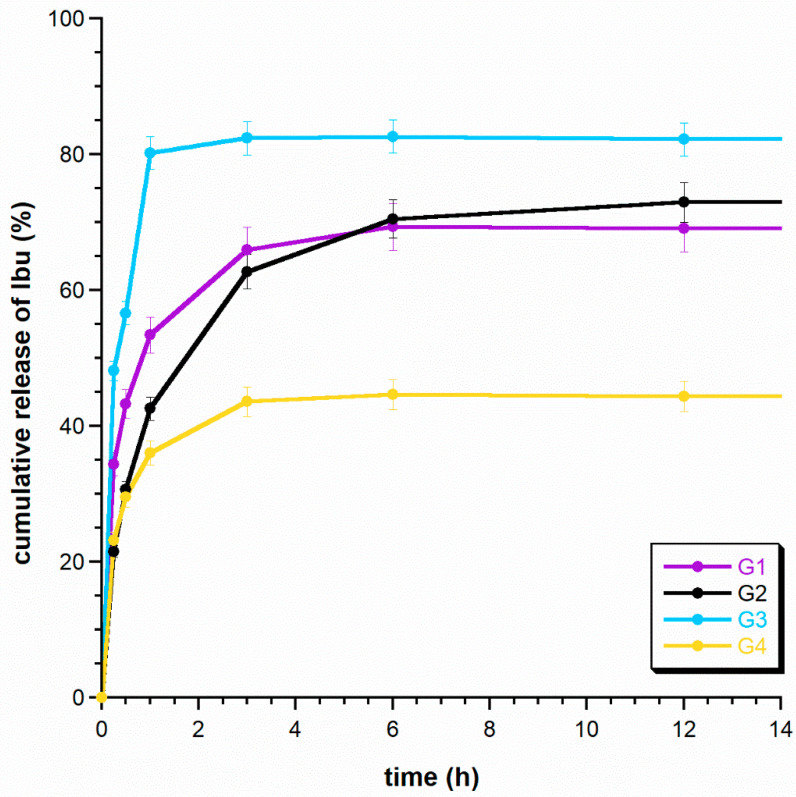
Results of the release of Ibuprofen from the granules.

Figure 5 shows the cumulative release curves of ibuprofen for the obtained granules. The graph shows the release up to 12 h, because no significant changes in the amount of drug released were observed thereafter. As expected, ibuprofen was rapidly released from all composite granules obtained within the first three hours and took the form of burst-release profiles. Each sample released more than 40% of the initially loaded drug within 3 h. It is worth paying attention to the G3 sample, where ibuprofen was released rapidly and, in fact, was over 80% released within 3 h. G1 and G3 had similar drug-release profiles over the studied time range. They showed the highest release effect extremely quickly, because after 1 h, the release reached 54% and 66% for G1 and G3, respectively. Samples G2 and G4 were characterized by slightly different curves. The release of Ibu was slightly slower—within 1 h, the release reached 43 and 36%, respectively. Interestingly, the maximum amount of drug released from the G4 sample during the tested 12 h was only 45%. It can be assumed that the reason for the slower release of ibuprofen may be the covering of the granules with chitosan, which significantly reduced their permeability during this time. These types of ibuprofen release profiles could be useful when an immediate high dose is required, such as in acute infections or inflammation [36,41]. We assume that a thicker chitosan coating with a greater degree of cross-linking could slow down the release of the drug, which would be beneficial, for example, when administering antibiotics [42].

**Figure 6:**
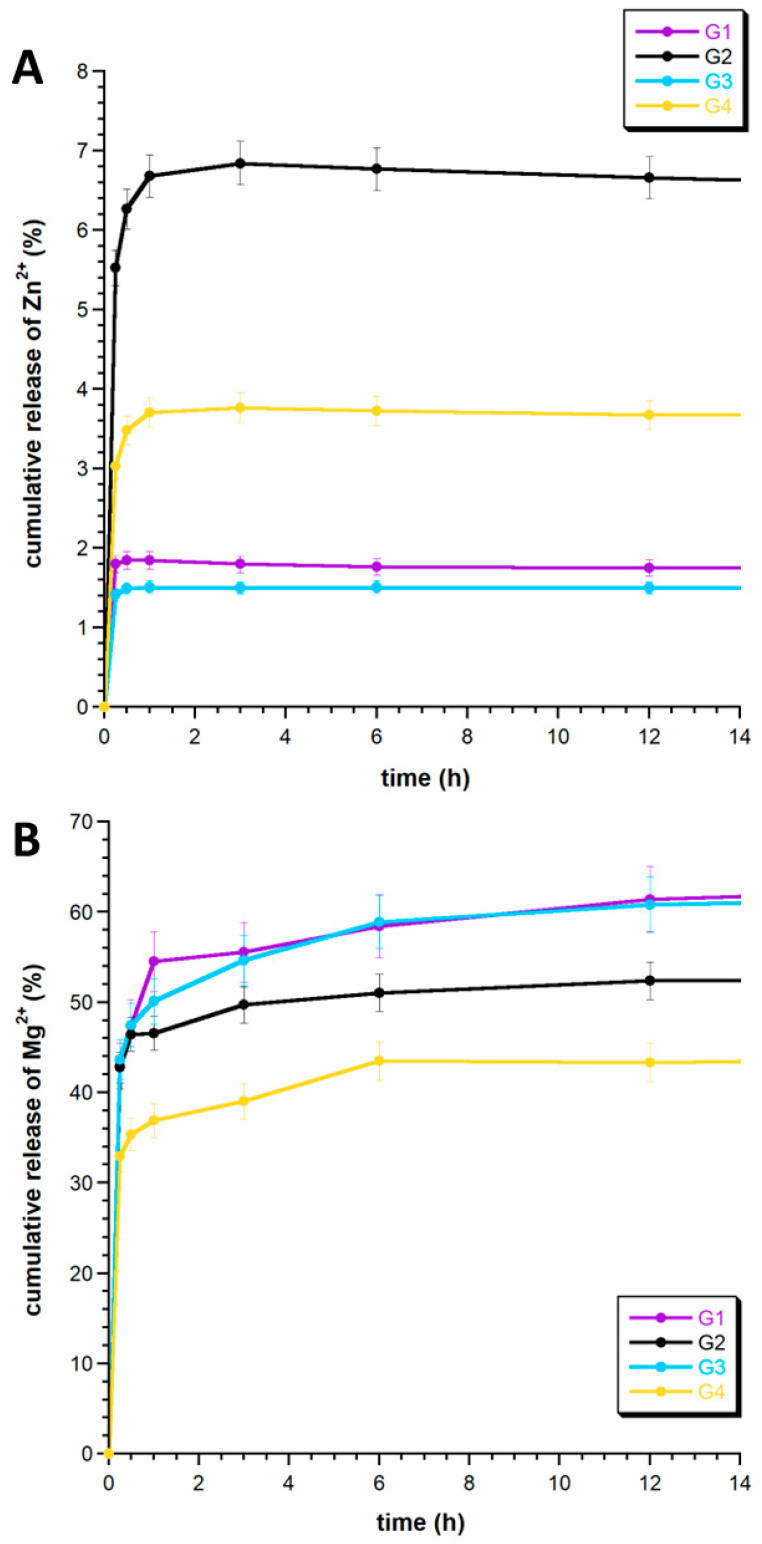
The release profiles of zinc ions (**A**) and magnesium ions (**B**).

In the next step, the release of zinc and magnesium ions, incorporated into the mineral phase of the granules (mHA), was executed. Cumulative release curves for the ions spanning a 12 h duration are delineated in Figure 6A,B. After 12 h, no significant changes in the release profile were observed. A pronounced disparity was evident in the release kinetics of magnesium and zinc ions across all granules, with magnesium exhibiting a notably higher degree of release in comparison to zinc.

This observation substantiates our previous hypothesis that magnesium ions undergo partial deposition on the hydrated surface layer of apatite. Consequently, this phenomenon facilitates their easier release from microcrystalline hydroxyapatite (mHA) and its composite materials [11]. For instance, in G1 and G3, approximately 60% of the magnesium ions were released within the 12 h timeframe, while for G2 and G4, this value was comparatively lower, at 52% and 43%, respectively. We posit that this discrepancy is associated with the presence of a chitosan coating [43].

Contrastingly, zinc ions predominantly reside within the crystalline apatitic structure, and cannot be completely released in such a limited time (12 h). In samples G1 and G3, the cumulative release did not surpass 2%, whereas in G2, a slightly higher release was observed, albeit not exceeding 7% within the stipulated period. It can be assumed that zinc ions will be released to a greater extent from the granules only after their degradation and the slow resorption of apatite.

### 2.6. Cytotoxicity Assay

Table 2 shows the results of the cytotoxicity assessment of the materials. No decrease in MG-63 cell viability was observed for any of the dilutions tested. The relative cell viability for the extracts was more than 70% of the untreated control and all tested materials were classified as non-cytotoxic.

The most widely recognized guidance documents for the biological safety evaluation of medical devices are the ISO 10993 series of standards developed by the International Organization for Standardization. The tested materials demonstrated safety in the in vitro cytotoxicity assessment test performed according to ISO 10993-5:2009. Cytotoxicity testing is a primary method to establish the safety of biomaterials. Although in vitro methods cannot reflect all the complexities present in the body during in vivo testing, they precede in vivo testing by predicting the potential behavior of a newly developed biomaterial in contact with tissue [44]. Further studies would require more biological tests, including in vivo tests [45].

It is noteworthy that protecting the material against bacterial biofilm formation is of paramount importance. The composite components were endowed with antibacterial properties, primarily sericin, which, as documented in the literature, has demonstrated efficacy against both Gram-positive and Gram-negative strains [46]. Furthermore, zinc ions are acknowledged for their bacterial growth-inhibiting properties and, notably, their ability to attenuate the development of bacterial biofilms [47]. Our future research will focus on assessing the antibacterial activity of the developed composites. Additionally, we will aim to investigate their potential as carriers for specific antibiotics, such as clindamycin or amoxicillin.

## 3. Materials and Methods

### 3.1. Synthesis of Zn^2+^-, Mg^2+^-, and CO_3_^2−^-Substituted Apatite Powder

Hydroxyapatite, mimicking bone apatite and enriched with zinc, magnesium, and carbonate ions, was synthesized using the conventional wet method (coprecipitation in an aqueous solution), which is described in detail in our previous work [11,48]. Calcium nitrate tetrahydrate (Ca(NO_3_)_2_∙4H_2_O), ammonium hydrogen phosphate ((NH_4_)_2_HPO_4_), diammonium carbonate ((NH_4_)_2_CO_3_), magnesium nitrate hexahydrate (Mg(NO_3_)_2_∙6H_2_O), and zinc nitrate hexahydrate (Zn(NO_3_)_2_·6H_2_O) served as the substrates for the synthesis. All the reagents were of analytical grade and purchased from Sigma Aldrich Chemicals, St. Louis, MO, USA.

An adequate amount of the reagents was weighed out to obtain a compound with a specific nominal composition: Ca_8,25_Zn_0,5_Mg_0,25_(PO_4_)_5_CO_3_OH. The sources of phosphates and carbonates were slowly dropped into the sources of calcium, zinc, and magnesium cations, stirring constantly. The pH was adjusted to about 11 using a concentrated ammonia solution. The slurry was left to age for 7 days without being stirred. After that, the precipitate was washed several times with distilled water, filtered, and dried at 100 °C for 24 h. Then, the obtained material was homogenized in an agate mortar. The powder was named mHA (mimetic hydroxyapatite).

### 3.2. Preparation of Composite Granules

In this work, four types of composite granules were obtained. The following reagents were used to fabricate the granules: atelocollagen from the bovine dermis (3 mg/mL) (Cosmo Bio Co., Ltd., Tokyo, Japan), sodium alginate (Sigma Aldrich, Burlington, MO, USA), anhydrous calcium chloride (CaCl_2_) (Sigma-Aldrich, Shanghai, China), ibuprofen sodium salt (Sigma Aldrich, Burlington, MO, USA), and the previously synthesized mHA powder. Some granules were enriched with silkworm sericin (Sigma Aldrich, Burlington, MO, USA) and chitosan (50–190 kDa molecular weight, 75–85% deacetylation degree, viscosity ≤ 300 cP, Sigma-Aldrich, Burlington, MO, USA).

Briefly, to prepare one matrix, 20 mL of atelocollagen from a bovine dermis was mixed with 10 mL of 3% alginate solution, and 840 mg of mHA was used. The suspension was mixed with a mechanical stirrer thoroughly until dense, and a milky slurry was acquired. Approximately 0.1 g of ibuprofen sodium (Sigma Aldrich, Burlington, MO, USA) was then added to each of the slurries. Four identical suspensions were obtained (G1-G4). Samples G3 and G4 were additionally enriched with 60 mg of sericin. At the same time, a cross-linking aqueous solution containing CaCl_2_ (1.5%) was prepared. Each of the slurries was added dropwise to the cross-linking solution with magnetic stirring, and granules were formed. The obtained granules were washed several times with distilled water, dried in air at room temperature, and lyophilized for 24 h. The type 2 and 4 granules (G2 and G4) were then coated for 30 min in a 0.5% chitosan solution. The chitosan was previously dissolved in 1% acetic acid. The chitosan-coated granules were quickly treated with 0.01 M NaOH, and later, they were rinsed carefully with deionized water. The G2 and G4 materials were lyophilized again for 24 h. All variants of the obtained scaffolds are presented in Table 3.

### 3.3. Analytical Methods

#### 3.3.1. Physicochemical Analysis of the mHA Powder

To validate the identity of the obtained hydroxyapatite (mHA), Fourier-transform infrared spectroscopy (FT-IR) was used with a PerkinElmer Spectrum 1000 spectrometer (Waltham, MA, USA). A transmission spectrum in the 4000–400 cm^−1^ range was obtained with a spectral resolution of 2 cm^−1^, employing KBr pellets and 30 scans. A phase composition analysis of the synthesized powder was performed using powder X-ray diffraction (PXRD). A Bruker DX8 Discover diffractometer (Billerica, MA, USA) with CuKα radiation (λ = 1.54 Å) within the 2 theta range from 20° to 70° was utilized. The microstructural features of the powder sample were scrutinized using a JEM 1400 transmission electron microscope (TEM-JEOL Co., Ltd., Tokyo, Japan, 2008) equipped with an 11-megapixel MORADA G2 TEM camera (EMSIS GmbH, Münster, Germany) under an accelerating voltage of 80 kV. The preparation of the analyzed material involved suspending the powder sample in 96% ethanol, followed by deposition onto a copper grid and subsequent air-drying. The measurements of the magnesium and zinc content were executed through inductively coupled plasma–optical emission spectroscopy (ICP-OES) using an Optima 3100 XL PerkinElmer spectrometer (Llantrisant, UK). The powder sample underwent dissolution in concentrated HNO_3_ (Suprapur, Sigma Aldrich, St. Louis, MO, USA) and an appropriate dilution with deionized water. The carbonate content (types A + B) was calculated using a methodology previously elucidated by Clasen and Ruyter [49].

#### 3.3.2. Physicochemical Analysis of the Composite Granules

The surface morphology of the composite granules was analyzed using a JEOL LTD. JSM-6390LV (Tokyo, Japan) scanning electron microscope (SEM) at an operating voltage of 20 or 30 kV. For the best contrast, the samples were sputter-coated with Au.

The porosity and specific surface area (S_BET_) of the G1, G2, G3, and G4 samples, the total pore volume (V_C_), and the distribution of their diameters (in the range of 2–300 nm) were determined from the N_2_ adsorption isotherm in the relative pressure range of p/p_0_ = 0.02–0.99 using the Brunauer–Emmett–Teller (BET) method with nitrogen adsorption (ASAP 2050 Micromeritics, Georgia, GA, USA).

A Shimadzu IRAffinity-1S transform infrared spectrometer (Kyoto, Japan) was used to measure the chemical structure of the samples. The granules were directly measured with an attenuated total reflection (ATR) accessory. The ATR-FTIR spectra were recorded in the 4000 cm^−1^–400 cm^−1^ range, at a spectral resolution of 2 cm^−1^, using 30 scans. All of the obtained spectra were processed using the GRAMS/AI 8.0 (Thermo Fisher Scientific, Waltham, MA, USA) and KaleidaGraph 3.5 (Synergy Software, Reading, PA, USA) software.

For the determination of swelling behavior, all of the obtained granules (G1, G2, G3, and G4) were incubated in ultrapure water at 37 °C. Subsequently, the samples were withdrawn from the solution after soaking for 15, 30, 60, or 180 min, and the adsorbed surface water was carefully removed using filter paper. The swelled samples were then weighed immediately. The swelling ratio was expressed as the percentage of increased weight (W-W0) to the initial weight (W0). Each sample was tested in triplicate.

The in vitro release of ibuprofen from the granules was evaluated in 50 mL Falcon tubes. The studies were performed in a phosphate buffer (pH = 7.4). Each tube contained 300 mg of a specific type of granule immersed in 50 mL of the release medium. The samples were placed in the bath shaker and stirred at 100 rpm at 37 °C before being incubated for seven days. Sample aliquots of 5 mL were withdrawn at regular time intervals (15 min, 30 min, 1 h, 3 h, 6 h, 12 h, 24 h, and 48 h). All the samples were filtered through a membrane syringe filter with a pore size of 0.8 μm. Each time, the volume of the medium taken for analysis was replaced with a new phosphate buffer portion. The samples were then analyzed using HPLC with chromatographic equipment, consisting of a Varian Prostar 210 isocratic pump (Palo Alto, CA, USA) and a Rheodyne 7725i injector (Cotati, CA, USA) with a 20 μL sample loop. Detection was performed using a Varian Prostar 325 UV detector with a detection wavelength of 220 nm. The chromatographic conditions and measurement procedures have been previously described [50]. The LC column used was a 4.6 mm i.d. × 250 mm length XTerra RP 18 analytical column that was purchased from Waters (Milford, Ireland). The mobile phase consisted of a phosphate buffer (pH of 7.0) mixed with acetonitrile in a ratio of 40:60, *v*/*v*. It was degassed using sonication before use. The flow rate of the mobile phase was maintained at 1 mL/min. The HPLC analysis was conducted at 30 °C. The peak areas were measured for the quantitation of the Ibu. Stock solutions of Ibu (1 mg/mL) were prepared by dissolving the appropriate amount in water. Calibration standards were prepared over a concentration range of 0.01, 0.025, 0.05, 0.1, 0.25, and 0.5 mg/mL for Ibu through appropriate dilutions of the above-mentioned standard solution. The calibration standards were analyzed in triplicate for the calibration curve.

The in vitro release of zinc and magnesium ions was studied in the same manner as the Ibu release test. The concentration of magnesium and zinc ions released into the PBS solution was determined with AAS using an AAnalyst 400 AA Spectrometer (Perkin Elmer, Waltham, MA, USA).

#### 3.3.3. Cytotoxicity Study

The cytotoxicity of the materials was evaluated according to the procedure in the ISO 10993-5:2009 guideline [51] using an MTT assay, with MG-63 cells. The MG-63 cell line, commercially available adherent human osteosarcoma cells, was purchased from ATTC, a commercial culture collection that distributes standard reference cell lines for research and development, with all the required certificates of quality and culture method specification. The test was performed using a cell exposure schedule on the extracts, with a readout technique based on reducing the MTT tetrazolium dye (3-(4,5-dimethylthiazol-2-yl)-2,5-diphenyltetrazolium bromide) to insoluble formazan in a microplate format. According to the ISO protocol, the volume of the extraction medium absorbed by each 100 mg of material was determined. This additional volume was then added to each 100 mg in the extraction mixture during the extraction of the material. The highest extract concentration tested was 50 mg/mL. The biomaterials were UV-sterilized before the preparation of the extracts. The extracts were prepared by incubating the samples in an MEM medium with 5% bovine serum for 24 h at 37 °C with stirring; the extracts were then sterilized by filtration. Before the treatments, the cells were seeded at a density of 1 × 10^5^ cells per well in 96-well microplates and were incubated for 24 h. Subsequently, the cells were treated with seven dilutions of each extract in a twofold dilution series for 24 h (50 mg/mL, 25 mg/mL, 20 mg/mL, 12.5 mg/mL, 10 mg/mL, 5 mg/mL, and 2.5 mg/mL). Following the exposure, the cells were washed with PBS and were incubated with an MTT solution for 2 h. The cells were then rinsed with PBS and isopropanol was added to dissolve the formed formazan. The absorbance of the solution in each well was measured at 560 nm in an Asys UVM340 Hightech microplate spectrophotometer. Highly cytotoxic latex and non-cytotoxic polyethylene foil were used as reference materials. The samples were considered cytotoxic if they reduced cell survival below 70% when compared to the untreated cells.

In our study, the experiments were performed in triplicate (*n* ≥ 3). The data are shown as mean values ± SD.

## 4. Conclusions

A composite of Zn^2+^-, Mg^2+^-, and CO_3_^2−^-substituted apatite and type I collagen was employed in the fabrication of granular forms serving as carriers for the model drug, ibuprofen. Alginate and/or sericin were used for granule formation, and in addition, chitosan was applied for coating. The resulting granules were characterized by an irregular, porous, and well-developed surface with mesopores. These granules exhibited high water absorption, with the chitosan coating contributing to a delayed absorption process. Ibuprofen demonstrated burst-release profiles, while distinct release mechanisms were observed for zinc and magnesium ions. All the obtained materials exhibited non-cytotoxic behavior at 24 h in the experiments.

Our preliminary research indicates the potential application of the obtained composites. Nevertheless, further comprehensive studies, including an analysis of microbiological activity, mechanical testing, and in vivo studies, are essential for a thorough understanding of their practical utility.

## Figures and Tables

**Table 1:** Porosity measurements of G1-G4 granules using the nitrogen-adsorption technique.

Sample	Surface Areaz S_BET_ (m^2^/g)	Pore Volume V_C_ (cm^3^/g)	Mesopore Volume V_mez._ (cm^3^/g)
G1	94	0.13	0.12
G2	79	0.21	0.18
G3	76	0.21	0.18
G4	85	0.23	0.21

**Table 2:** Results of the MTT assay for the highest concentrations of tested extracts (50 mg/mL) in comparison to the untreated control. LT—latex, reference cytotoxic material. PE—polyethylene foil, reference non-cytotoxic material.

Sample	Cell Viability ± SD (%)	IC50 (mg/mL)	Classification
G1	92 ± 15	N	Non-cytotoxic
G2	103 ± 14	N	Non-cytotoxic
G3	92 ± 12	N	Non-cytotoxic
G4	83 ± 9	N	Non-cytotoxic
LT	1 ± 1	8	Cytotoxic
PE	102 ± 9	N	Non-cytotoxic

**Table 3:** Composition of the granules.

Type of Granule	Atelocollagen	Sodium Alginate	mHA	Sericin	Chitosan	Ibuprofen
G1	+	+	+	-	-	+
G2	+	+	+	-	+	+
G3	+	+	+	+	-	+
G4	+	+	+	+	+	+

## Data Availability

Data is contained within the article.
